# Healthcare Providers’ Perceptions of the Cancer Pain Management Barriers at a Hospital in Zambia: A Qualitative Study

**DOI:** 10.1177/23779608231197008

**Published:** 2023-09-03

**Authors:** Jennipher Kombe Mulonda, Yolanda Havenga, Martjie de Villiers

**Affiliations:** 56412 Adelaide Tambo School of Nursing Science, Tshwane University of Technology, Pretoria, South Africa

**Keywords:** barriers, cancer, healthcare provider, hospital, management, pain, perception, Zambia

## Abstract

**Introduction:**

Cancer incidence and mortality are increasing worldwide, and pain is the most common symptom patients experience. Despite developments in cancer pain management and guidelines, the pain often remains undertreated. Effective pain management ultimately involves overcoming several complex institutional, patient, and healthcare provider-related barriers.

**Objective:**

The objective of the study was to explore and describe healthcare providers’ perceptions of the cancer pain management barriers at a hospital in Zambia.

**Method:**

A descriptive qualitative study was conducted. Data were collected from 10 purposively sampled medical doctors and registered nurses using prepiloted semistructured individual interviews. Interviews were audio recorded and transcribed verbatim. Thematic analysis was done, and trustworthiness was enhanced based on the criteria described by Lincoln and Guba. Ethical principles, as outlined in the Declaration of Helsinki, were adhered to.

**Results:**

Three themes emerged, namely patient-related barriers (biographic characteristics, patient knowledge and perceptions), healthcare provider-related barriers (knowledge and perceptions and language barriers), and institution-related barriers (resource limitations and lack of standards and guidelines). Healthcare providers’ views that cultural beliefs about cancer being caused by witchcraft and the use of traditional medicine and services are barriers to cancer pain management were unique to this study.

**Conclusion:**

Cancer pain management requires a total pain management approach that addresses the barriers to pain management strategies from a patient, healthcare provider, and institutional perspective. The knowledge deficit among patients and healthcare providers is a barrier to cancer pain management and one of the most common challenges reported in the literature. This study further points toward a need to develop culturally competent healthcare providers.

## Introduction

Cancer incidence and mortality are increasing worldwide, with pain being the most common symptom experienced by patients (Jara et al., 2018). However, cancer pain is poorly managed in resource-scare settings, leading to preventable suffering (Kettyle, 2023; WHO, 2018). Several studies (Goucke & Chaudakshetrin, 2018; Kwon, 2014) reported that effective pain management means overcoming several complex barriers categorized as institutional, patient, and healthcare provider-related (Uysal, 2018).

## Review of Literature

Cancer incidence and mortality are global concerns. In 2020, 19.3 million patients were newly diagnosed, and 10 million died of cancer (Sung et al., 2021). The global cancer burden is expected to affect 28.4 million people by 2040, a 47% increase from 2020, with the increase more considerable in transitioning than in transitioned countries (Sung et al., 2021). The Zambia National Cancer Registry (Ministry of Health Zambia, 2018) reported that in a population of 17 million people, 12,052 patients were newly diagnosed with cancer, and there were 7,380 cancer-related deaths.

Pain is experienced by 55% of patients receiving anticancer treatment, 66% of patients with an advanced, metastatic or terminal disease (van den Beuken-van Everdingen et al., 2016), and 10% of cancer survivors in the United States of America (Gallaway et al., 2020). Pain is a multidimensional concept attributed to physiological mechanisms that cause unpleasant sensory and emotional experiences associated with actual or potential tissue damage, classified as nociceptive or neuropathic (Fallon et al., 2018; Kettyle, 2023; WHO, 2018). Psychological, social, and spiritual factors compound physical pain, called a “total pain” experience (Kettyle, 2023; WHO, 2018). Moreover, pain occurs with additional symptoms such as fatigue, sleep disturbances, loss of appetite, and anxiety that affects patients’ quality of life, physical functioning, social relationships, and mental health (Salim et al., 2017).

Pain relief is necessary at all stages of cancer (WHO, 2018), and better results are obtained when pain management is introduced earlier in the course of the illness and combined with disease-focused therapies. Furthermore, pain management should be patient-centred (Haun et al., 2017). Patient-centred pain management requires a holistic approach based on the assumptions of the “total pain” model (Mehta & Chan, 2008). Understanding and implementing the model during pain assessment and management aids in understanding the patient's total pain experience and promotes optimal pain management by healthcare providers (Mehta & Chan, 2008).

The backbone of cancer pain treatment is pharmacological interventions. However, other therapies should be included in a holistic approach to managing pain, namely radiotherapeutic, anesthetic, neurosurgical, psychological, physical therapy, and spiritual and social interventions (Scarborough & Smith, 2018; WHO, 2018). Cancer pain management thus requires a multidimensional (Javier et al., 2016; Kwon, 2014) and multidisciplinary approach (Uysal, 2018).

However, despite developments in cancer pain management and guidelines, the pain often remains undertreated (Jara et al., 2018), with up to 31% of patients’ cancer pain not effectively controlled (Breivik et al., 2009). Ineffective cancer pain management is worse in developing countries, where treatment coverage is limited, and many patients with cancer do not receive appropriate pain management (Ahmedzai et al., 2019; Makhlouf et al., 2023; Nia & Epstein, 2019). In these resource-limited countries, up to 80% of patients dying from cancer experience moderate to severe pain lasting, on average, for up to 90 days (Knaul et al., 2018).

Several barriers to effective pain management have been identified. These barriers are context-specific but can be broadly categorized as patient-related, healthcare provider-related and institution-related (Goucke & Chaudakshetrin, 2018; Kwon, 2014). Patient-related barriers include cognitive, affective and adherence-related factors, negative beliefs and fears related to using analgesics (Kwon, 2014). Healthcare provider-related factors include a lack of knowledge and skill to assess and manage cancer pain, misperceptions about the use of analgesics, inadequate assessments of a patient's pain level, and ignorance of existing standard pain assessment scales (Goucke & Chaudakshetrin, 2018; Kwon, 2014; Omran et al., 2014; Saifan et al., 2019; Uysal, 2018; Yu et al., 2017). Institutional barriers include limited access to opioids and specialist treatment, country-specific regulatory requirements in the prescription of opioids, limited resources, especially in rural and remote areas, and a lack of available guidelines (Goucke & Chaudakshetrin, 2018; Kwon, 2014; Sharkey et al., 2018).

In Zambia, studies by Kalolo (2010) and Mutale et al. (2018) on cancer pain management indicate that patients with cancer do not always receive appropriate medication because of poor pain assessments and the unavailability of pain management drugs. However, relieving all forms of pain and suffering is an ethical duty of healthcare providers and a human right (Carvalho et al., 2018). The obstacles to cancer pain management should thus be overcome, and interventions must be developed to optimally manage this population's pain (Jara et al., 2018; Uysal, 2018). Due to limitations in the management of pain in the context of this study, healthcare providers’ perceived barriers regarding cancer pain management at a hospital in Zambia were explored and described to address these barriers holistically.

## Methods

### Design

A descriptive qualitative design (Polit & Beck, 2021) explored and described cancer pain management barriers in this context. Semistructured interviews were conducted with a purposively sampled group of professional nurses and medical doctors working at a specific hospital in Zambia. The descriptive qualitative design enabled an exploration of meaning and understanding (Creswell & Creswell, 2018; Polit & Beck, 2021) of the barriers to cancer pain management healthcare providers reported in an inductive way, creating the possibility of determining whether any other barriers not explained in literature might be present in this study's setting.

The hospital functions as an outpatient treatment facility that offers radiation therapy, chemotherapy, hormonal treatment and palliative care to patients with cancer. It is a government grant-aided hospital, and at the time of data collection, treatment was free for all Zambians. The hospital serves as a national referral centre for patients with cancer nationwide, with a catchment population of approximately 17 million, covering the ten provinces of Zambia. In addition, the hospital also caters to patients from neighbouring countries, including Malawi, the Democratic Republic of Congo and Zimbabwe. When patients require admission, they are referred to a nearby hospital.

### Research Questions

The study had one research question: What are the barriers to cancer pain management at a specific hospital in Zambia? The subquestions were: What are the patient-related barriers to cancer pain management? What are the healthcare provider-related barriers to cancer pain management? What are the institution-related barriers to cancer pain management?

### Sample

The population consisted of all healthcare providers working at the hospital, which included 38 registered nurses and 12 medical doctors. Purposive sampling (Polit & Beck, 2021) was used to select participants based on the following inclusion criteria: professional nurses and medical doctors with at least 6 months of experience working at the specific hospital. The sample size was not predetermined but was based on the data saturation principle (Polit & Beck, 2021). The sample included 10 healthcare providers. Saturation was reached with the seventh participant, but three additional interviews were conducted to confirm no new themes.

### Data Collection

**Figure 1.**
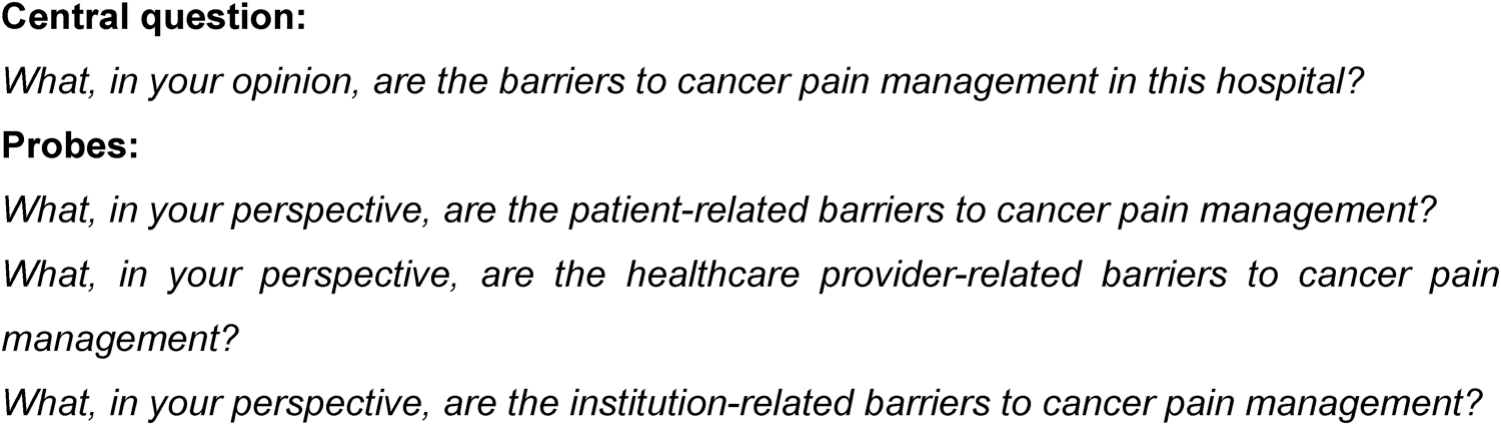
Interview schedule.

Data were collected from June 2017 to December 2017 using semistructured individual interviews based on an interview schedule. The interview schedule was piloted with three participants, and information from the pilots was not included in the presented data. The semistructured individual interviews were opened with a broad central question. After that, probes were used to focus on the patient, healthcare provider and healthcare institution. The interview questions are included in Figure 1. The interviews were conducted in a private room, audio recorded, and field notes were kept. The researcher applied effective recommended interviewing skills while conducting the semistructured interviews (Creswell & Creswell, 2018).

### Data Analysis

Data were thematically analysed (Polit & Beck, 2021). First, audio-recorded interviews were transcribed verbatim and organized with field notes in the corresponding text. Interviews were then read through and coded one by one. The three probing questions about patient-related, healthcare provider-related and institution-related barriers to cancer pain management formed the three themes from which categories and subcategories were identified. The findings were described using verbatim quotes from participants to support the categories.

### Trustworthiness

Lincoln and Guba's (in Polit & Beck, 2021) criteria of credibility, transferability, confirmability, dependability, and authenticity were enhanced by the following activities:

- Spending adequate time with participants and establishing rapport;
- Providing dense descriptions of the research methodology and the findings;
- Purposively sampling participants who were knowledgeable about cancer pain management and who were working in the specific setting;
- Keeping an audit trail;
- Co-coding and reaching a consensus about the subcategories with an independent coder;
- Audio-recording interviews and using verbatim transcriptions; and
- Participant triangulation, including doctors and nurses.

### Ethical Considerations

Ethical approval to conduct the study was sought from the Tshwane University of Technology Research Ethics Committee [Ref #: FCRE 2016/04/002 (SCI)(2)] and Eres Converge IRB [Ref #: 2016-Oct-006]. Gatekeeper permission was given by the chief executive officer of the hospital where the research took place. Interviews were conducted during break times or after work to prevent the research from impacting service delivery. Written informed consent was also obtained from each participant before data collection, and participation was voluntary. Confidentiality and anonymity were ensured by anonymising the hospital and using codes for interviews and participants. Electronic data were password protected, and hard copies were securely stored for 5 years and then destroyed.

## Results

The results focused on the sample's characteristics and perceived barriers to cancer pain management.

### Sample Characteristics

Ten participants, including seven professional nurses and three medical doctors, participated in the study. Two participants had prior specialized training in oncology; one oncology-specialized medical doctor and one oncology-specialized professional nurse. Participants’ experience as healthcare providers ranged between 2 and 17 years, while years of experience in oncology ranged between 2 and 11 years.

### Barriers to Cancer Pain Management Results

**Figure 2.**
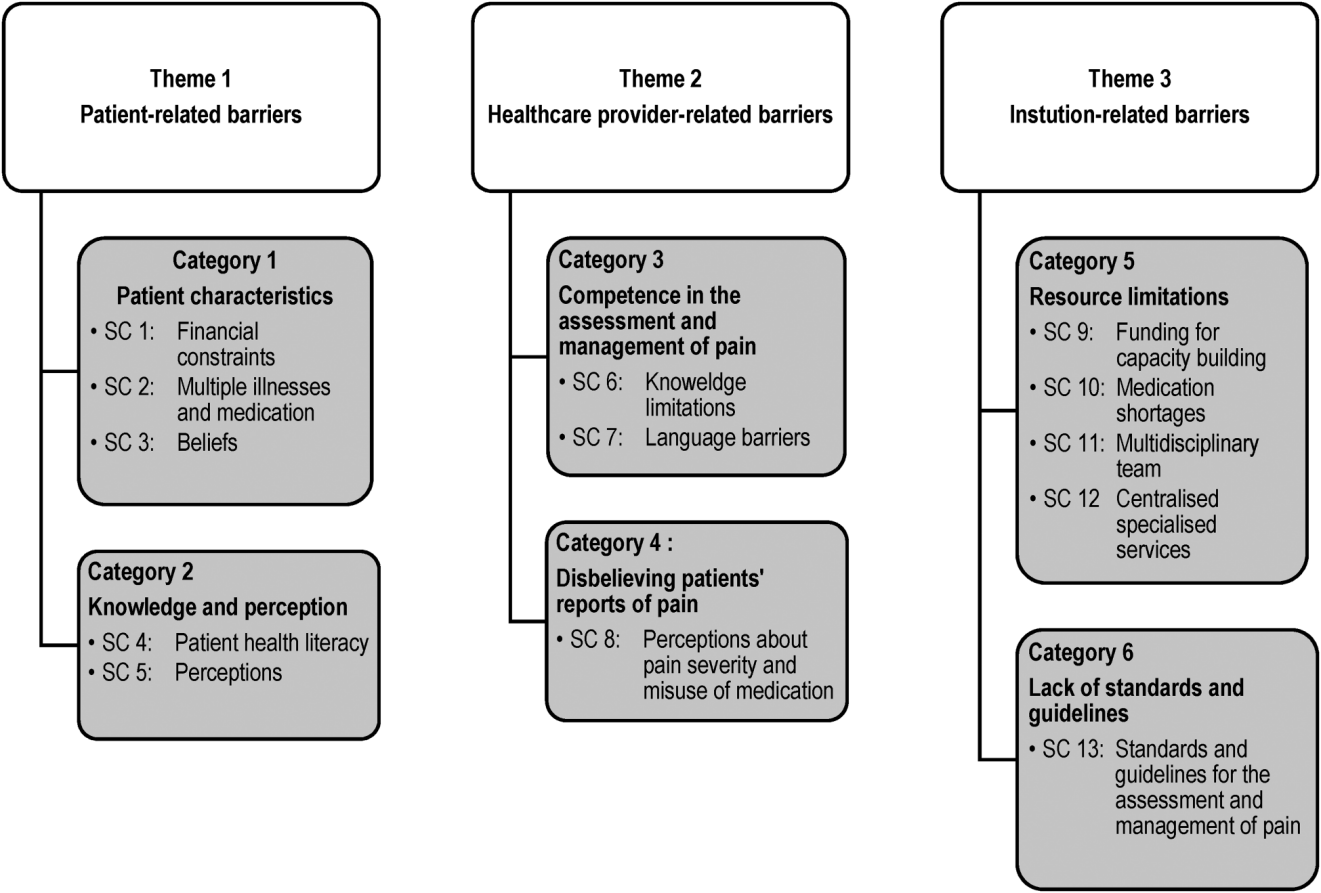
Themes, categories and subcategories.

Patient-related, healthcare provider-related, and institution-related barriers were identified and discussed. The three themes yielded six categories and 16 subcategories (SC), as outlined in Figure 2.

#### Theme 1: Patient-Related Barriers to Cancer Pain Management

Two patient-related barriers were identified: barriers related to patients’ biographic characteristics and patients’ knowledge and perceptions of cancer pain and pain management.

##### Category 1: Patient Characteristics

The perceived barriers related to patients’ biographic characteristics included financial constraints, multiple illnesses, the use of multiple medications, and patient beliefs.

Participants explained that patients who experienced financial constraints (SC 1) lacked the financial means for transport, resulting in them not honouring their appointments at the hospital and constraining their ability to adhere to treatment. The following statements supported this finding:

*Some patients would rather use the little money they have on food and daily expenses than use it for transport to come for a refill of pain medication.* (Nurse)

*Patients and their relatives must travel long distances to access the specialized service.* (Doctor)

Managing multiple medications also led to confusion about the correct use among patients and caregivers. Participants indicated that multiple medications often contributed to nonadherence to prescribed pain medication. Some participants mentioned that having multiple chronic diseases (SC 2) in addition to cancer led to patients taking several types of medication. The following statement reflects this subcategory:

*Because some patients have many more illnesses apart from cancer, they tend to have many more drugs to take, and it is confusing for them to know which drugs to take and when.* (Nurse)

Participants further described patients’ beliefs (SC 3) that cancer was caused by witchcraft as a barrier to pain management as patients often consulted traditional healers and preferred using their herbal medication to the prescribed pain medication. Participants were of the view that pain management was consequently compromised. One participant explained:

*Patients who have cancer pain turn to traditional healers. They believe their illness is caused by witchcraft. Hence, they are more comfortable seeing the traditional healer because they think he understands their problem better than the doctors at the hospital*. *Some patients think only the herbal medications can help in cases where they have severe pain, so they will just be taking home remedies for their cancer pain.* (Nurse)

Also, regarding gender (SC 3), it is difficult to obtain an accurate report of pain from male patients as there is a belief that an acknowledgement of pain is a sign of weakness, not befitting men. Participants explained that this notion prevented men from reporting pain, resulting in poorly managed cancer pain. The following quotes reflect this cultural belief:

*Because of traditions, culture and beliefs most male cancer patients will not tell you they are in pain … pain is not supposed to be announced or publicized … in our culture most male patients will not tell you that they are in pain.* (Nurse)

*When a man complains of pain, it is seen as a sign of weakness.* (Nurse)

##### Category 2: Knowledge and Perception

Healthcare providers explained that patients’ limited health literacy (SC 4) was a barrier to cancer pain management. Participants also stated that patients’ failure to read the instructions on the prescriptions led to incorrect use of prescribed pain medication. Furthermore, limited comprehension of the meaning of specific instructions, usage, storage, and the expiration of the drugs negatively impacted patients’ understanding of healthcare providers’ explanations. In the view of healthcare providers, illiteracy had a bearing on how patients would interpret the cause of pain, how it could be relieved, how analgesics work, and the cancer disease process. Participants explained:

…*they will formulate their instructions or make up any timings and dosing that suits them because they cannot read.* (Nurse)

*Some patients cannot read; they cannot even tell if the drug has expired or not* … *they will, in fact, even mix the old medicines with the one they have just gotten recently. They cannot distinguish which is a new or expired drug*. (Nurse)

Participants explained that patients perceived (SC 5) pain to be acceptable as this was to be expected when diagnosed with cancer. Patients, therefore, felt that it was not necessary to report their pain:

*Most cancer patients think pain is part of the disease, so they will not tell you they are in pain, … they think it is okay to have pain if you have cancer, … Patients think experiencing pain is part of the disease process.* (Doctor)

Participants expressed the view that patients attached certain myths to cancer pain management and how the drugs intended to relieve their pain instead caused them harm. Participants explained:

*Some patients have misconceptions that strong opioids will kill them instead of relieving the pain.* (Nurse)

*…some patients think that taking morphine will cause cancer to become resistant to chemotherapy or radiotherapy.* (Nurse)

#### Theme 2: Healthcare Provider-Related Barriers to Cancer Pain Management

Two categories of barriers related to cancer pain management were derived from this theme: competence in the assessment and management of pain, and disbelieving patients’ reports of pain.

##### Category 3: Competence in the Assessment and Management of Pain

Due to limited specialized training, healthcare providers experienced challenges in knowing (SC 6) which drug combinations worked well with various types of pain and how one drug could replace another while maintaining the same and safe desired effect. This finding was expressed as follows:

*Some health care providers do not know how to combine certain drugs to maximize the effect of pain relief.* (Nurse)

*We have the pain assessment forms, but using them confidently is difficult.* (Nurse)

*The way I would manage a headache caused by malaria is different from the way I would manage a headache caused by a brain tumour.* (Doctor)

Some participants reported that despite knowing about cancer pain management, they lacked confidence and feared being questioned about their practice:

*Healthcare providers have challenges in deciding what to prescribe to the patient to help relieve the cancer pain … health care providers sometimes unable to confidently decide on how to classify, grade and resolve the patients’ pain.* (Nurse)

Participants also mentioned that Zambia's multiple local and seven officially recognized languages resulted in language barriers (SC 7) that led to communication breakdowns. Participants explained:

*I may be a Zambian but can only fluently speak and understand 2 to 3 languages, worse still is when it comes to translation … Some patients are unable to describe the pain so that it can be graded.”* (Nurse)

*… some local languages do not have the grades to say mild, moderate or severe.* (Nurse)

##### Category 4: Disbelieving Patients’ Reports of Pain

Healthcare providers perceived patients misrepresent the severity of their pain, and they believed pain medication was misused (SC 8). Participants explained that they often did not believe patients’ reports of the pain experience (SC 8):

*As health care providers, we always want to look for the pain in the patient instead of listening to what the patient has to say* (Nurse)

*… health care providers being the experts in health-related issues, do not want to listen to what the patient has to say, and if they do not listen, how much pain they are experiencing, then it is difficult to manage the cancer pain because what gauge will they use?* (Doctor)

#### Theme 3: Institution-Related Barriers to Cancer Pain Management

Two institution-related barriers were identified: resource limitations, and a lack of standards and guidelines for pain management.

##### Category 5: Resource Limitations

Participants indicated that there needed to be more funding for healthcare providers’ capacity-building (SC 9). They expressed the value of specialized training in improving the quality of health services provided to patients at a specialized hospital as follows:

*With specialized training, our patients can receive the quality care they deserve.* (Doctor)

*Enough funding should be allocated for specialized training, for example, palliative care, oncology.* (Nurse)

Participants also mentioned medication shortages (SC 10) as a barrier to cancer pain management:

*We have limited types of pain medication … Certain cancer pains are complex and need complex drug combinations, but we are failing our patients in that regard* (Doctor)

In addition, participants explained that due to the complexity of cancer pain, it should be managed by a multidisciplinary team; however, in this context, there were a limited number of multidisciplinary team members (SC 11) involved in treating patients with cancer. As explained:

*Cancer is complex, and we need a completely specialized team to manage it.* (Doctor)

Participants also indicated that patients could not access specialized services as they were centralized at one hospital (SC 12):

*Our poor patients do not have the means to be moving back and forth to this distant hospital just for pain medication.* (Nurse)

*The government need to build more of such hospitals in various country locations to carter for the majority.* (Doctor)

##### Category 6 Lack of Standards and Guidelines

Some participants expressed concern about the hospital's lack of standards and guidelines for assessing and managing cancer pain (SC 13). Guidelines are crucial and impact uniformity in treating patients with cancer when they are in pain. Participants shared that:

*As a hospital, we do not have standards and guidelines that suit our setting and drug availability levels …. Each one prescribes according to their level of knowledge and experience.* (Doctor)

*What we are lacking is displayed standards and treatment guidelines in pain control, internationally recognized standards and guidelines like the World Health Organisation on cancer pain management which may need to be adopted and adapted to our setting.* (Nurse)

## Discussion

Based on the study's objective to explore and describe healthcare providers’ perceptions of the cancer pain management barriers at a hospital in Zambia, three themes emerged: patient-related barriers, healthcare provider-related barriers and institution-related barriers. The study's results reflect the multidimensional nature of barriers to cancer pain management, and these were also found in similar studies in a variety of other settings (Ahmedzai et al., 2019; Goucke & Chaudakshetrin, 2018; Samara et al., 2018; Toba et al., 2019).

### Patient-Related Barriers

In this study, healthcare providers perceived patients’ specific characteristics, namely their financial constraints, multiple illnesses, medications, and beliefs, as barriers to cancer pain management. Financial constraints affect patients’ ability to travel to healthcare facilities, negatively impacting the continuity of care. The findings that compliance with cancer treatment was related to the distance patients had to travel and the high costs associated with the travel and services are confirmed by other studies (Kidayi et al., 2023; Rick et al., 2021).

In this study, managing multiple illnesses with various medications impacted adherence to cancer pain management regimens. Similarly, Uysal (2018) stated that patients’ medical history and associated symptoms of other illnesses affected coping skills that enabled them to manage their pain.

Patients’ beliefs also affect their feelings about cancer pain management and, in turn, their behavior. In this study, two beliefs affecting adherence to cancer pain management regimens and follow-up included the belief that witchcraft was responsible for their cancer, and male patients not reporting pain due to the belief that this reflects weakness. Makhlouf et al. (2023) and Uysal (2018) also reported cultural beliefs as barriers to cancer pain management. In a study by Kwon (2014), negative attitudes toward morphine was reported, due to the fear of addiction, religious concerns, and cultural prohibitions. In addition to what was found in this study, Uysal (2018) reported a fatalistic approach to cancer pain management was driven by patients’ belief that chronic pain was unavoidable and unmanageable.

Furthermore, participants’ knowledge was a barrier to cancer treatment. Limited knowledge and misconceptions about pain medications lead to reluctance to report pain and adhere to pain regimens. Toba et al. (2019) similarly found that patients’ knowledge deficits were a barrier. Accordingly, Kwon (2014) states that a lack of knowledge among cancer patients will likely lead to miscommunication with healthcare providers, fear of addiction and fatalistic beliefs. In addition to patient knowledge, literature on the subject has shown that pain-related knowledge, attitudes, and beliefs among family caregivers interfere with the inherent complexity of cancer pain management, sometimes exacerbating patients’ difficulties (Meeker et al., 2011). Addressing patients’ and their families’ knowledge is an essential first step in the pain management process (Uysal, 2018).

### Healthcare Provider-Related Barriers

A perceived healthcare provider barrier was a lack of knowledge and skills in assessing and managing cancer pain. Various studies found that nurses and doctors did not have adequate knowledge about pain assessment and management (Nega et al., 2014; Omran et al., 2014; Toba et al., 2019). Javier et al. (2016) and Toba et al. (2019) determined that inadequate pain assessment by medical staff, which may be due to inadequate knowledge, an underestimation of the patient's complaint of pain, or inadequate communication and listening skills, were significant barriers that prevent the adequate control of cancer-related pain. Javier et al. (2016) further assert that time constraints and insufficient knowledge regarding pain management among medical professionals are the most commonly encountered barriers to effective pain management by both physicians and nurses.

Precise assessments and reassessments of the severity, type and cause of pain are essential in developing and implementing a holistic management plan to relieve cancer pain (Kettyle, 2023). Furthermore, for healthcare providers to apply holistic and multidisciplinary approaches to cancer pain management, they require knowledge about pharmacological and nonpharmacological approaches (Kettyle, 2023; Uysal, 2018; WHO, 2018).

Another finding in this study was that healthcare providers tended to disbelieve patients’ reports of pain. This finding is comparable to those from a previous study, wherein doctors tended to believe that patients overreported their pain more than nurses; as a result, patients complaining of pain were given a placebo (Nega et al., 2014).

Language limitations were another barrier to cancer pain management in this study. Inadequate communication and listening to patients can lead to adverse psychological outcomes, including dissatisfaction with care, increased anxiety, long-term maladjustment, and an unmet need for symptom management (Walling et al., 2016). In order to optimally assess patients’ pain based on the “total pain” model, clear communication is required (Mehta & Chan, 2008). These communication factors may further be related to cultural barriers or a lack of cultural sensitivity (Walling et al., 2016).

### Health Institution-Related Barriers

Barriers related to limited cancer pain management strategies and guidelines, and a lack of available medication, were mentioned in this study. Javier et al. (2016) concurred that a lack of available guidelines was one of the institution-related barriers to cancer pain management. A study by Makhlouf et al. (2023) also confirmed that healthcare providers perceived the absence of policies, guidelines, education and training in managing pain as a barrier to cancer pain management. This study confirmed that limited medications were available to manage cancer pain. Sharkey et al. (2018) found that in 2015, only 43% of countries reported that essential opioids were generally available in primary healthcare facilities in the public sector. This availability was significantly lower in low- and lower-middle-income countries. In addition to the limited guidelines and medication found in this study, Kwon (2014) asserted that healthcare providers’ capacity-building in cancer pain management was impeded by a lack of funding and equipment.

## Strengths and Limitations

A strength of the study was that medical doctors and nurses were included in the research sample, leading to the inclusion of the perspective of two categories of healthcare providers and, in so doing, triangulating findings. The inclusion of other healthcare providers’ views would have been valuable to gain a broader perspective of the barriers to holistic and comprehensive pain management.

## Implications for Practice

Healthcare providers’ knowledge limitations in the assessment and holistic management of cancer pain require education, training and ongoing capacity-building interventions. Multidisciplinary approaches to cancer pain management should include a culturally sensitive approach that acknowledges patients’ and their families’ beliefs about the cause of cancer, reporting of cancer pain, consequences of opioid use, and complementary and traditional medicine and practices. Furthermore, healthcare providers should be empowered to optimally use existing pain management methods and medications within the resource-constrained environment. Guidelines should also be context-specifically formulated to accommodate resource constraints. Ongoing advocacy for patients’ right to access resources and services to optimally manage cancer pain is required.

## Conclusion

Effective management of cancer pain can prevent suffering and improve patients’ quality of life. Cancer pain management requires a total pain management approach that addresses the barriers to pain management from a patient, healthcare provider, and institutional perspective. The knowledge deficit among patients and healthcare providers is a barrier to cancer pain management and is one of the most common challenges reported in the literature. Various approaches are required to educate patients, families and society about cancer pain management to reach a wider group. Healthcare provider education, during their studies and ongoing after completion of training, is essential to ensure adequate pain assessment and management strategies. Healthcare providers’ views that cultural beliefs about cancer being caused by witchcraft and the use of traditional medicine and services are barriers to cancer pain management were unique to this study. Advocacy for the availability of cancer pain management as a fundamental human right, also in resource-scarce environments, should be ongoing. The barriers should receive attention in a contextual plan to improve health providers’ management of cancer pain in the specific hospital in Zambia.
